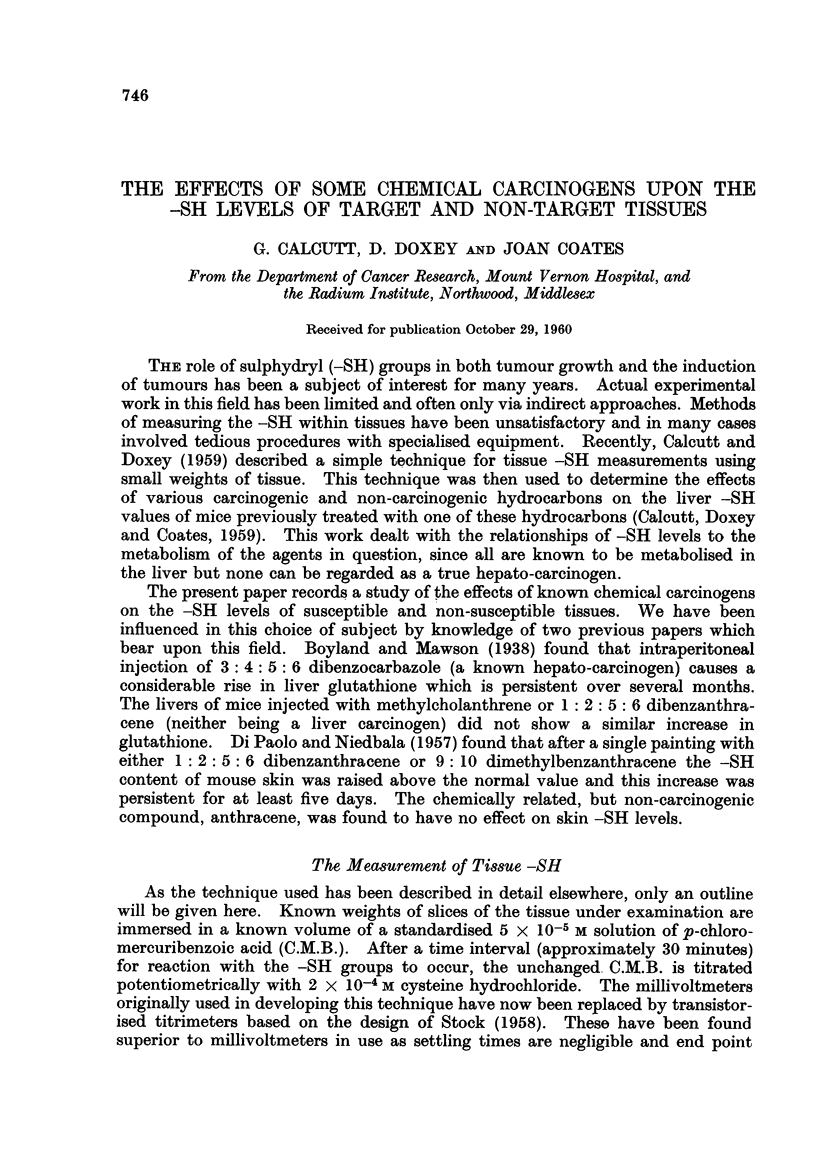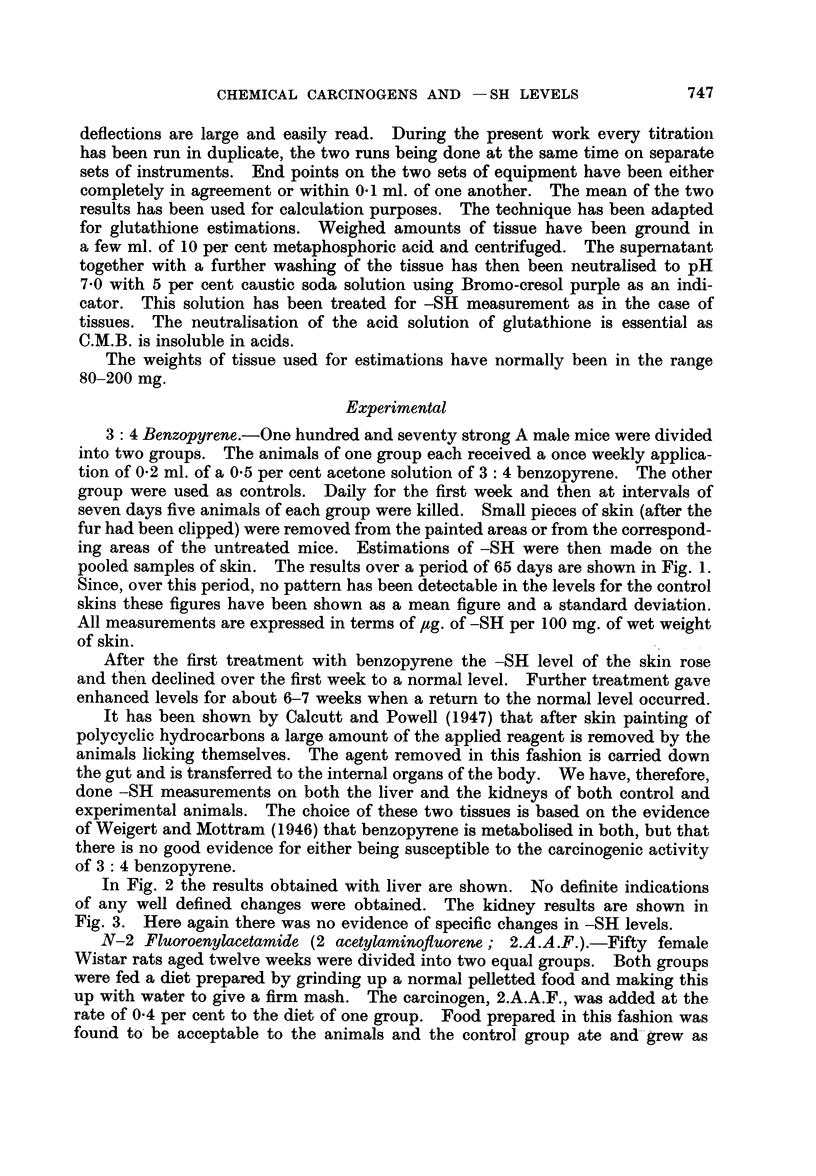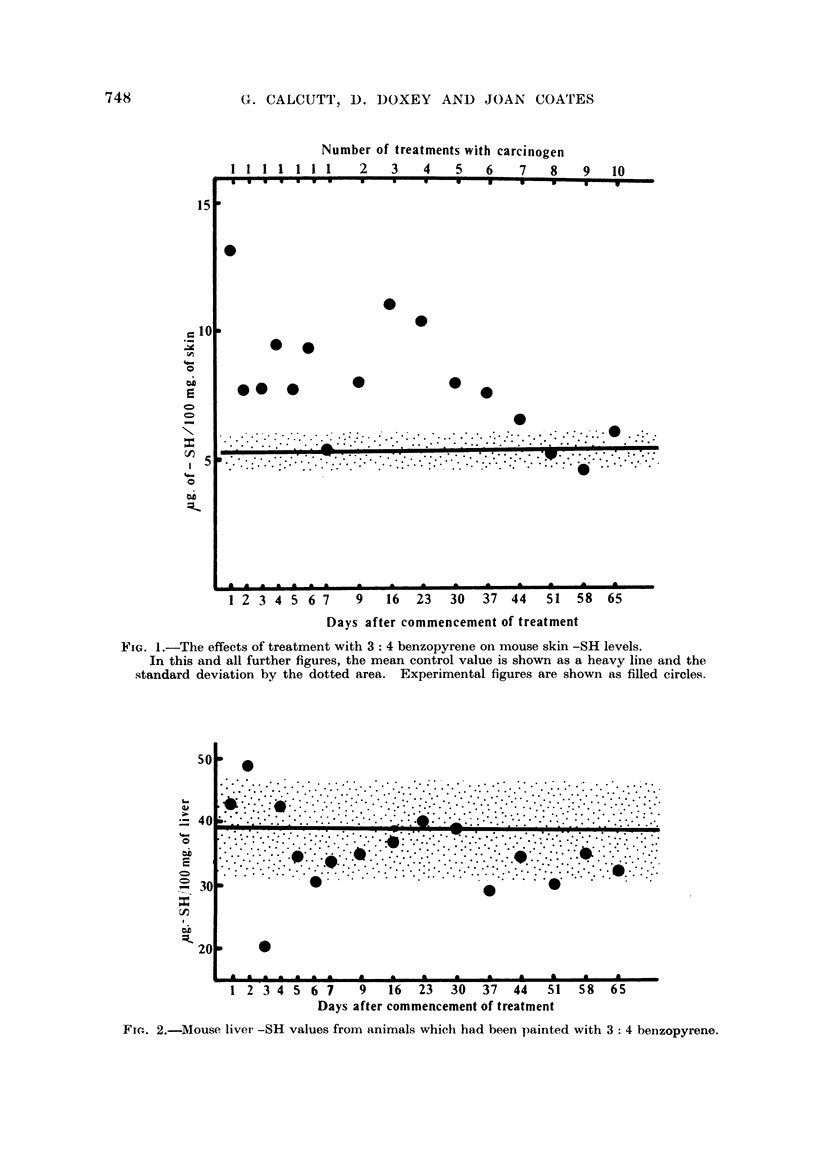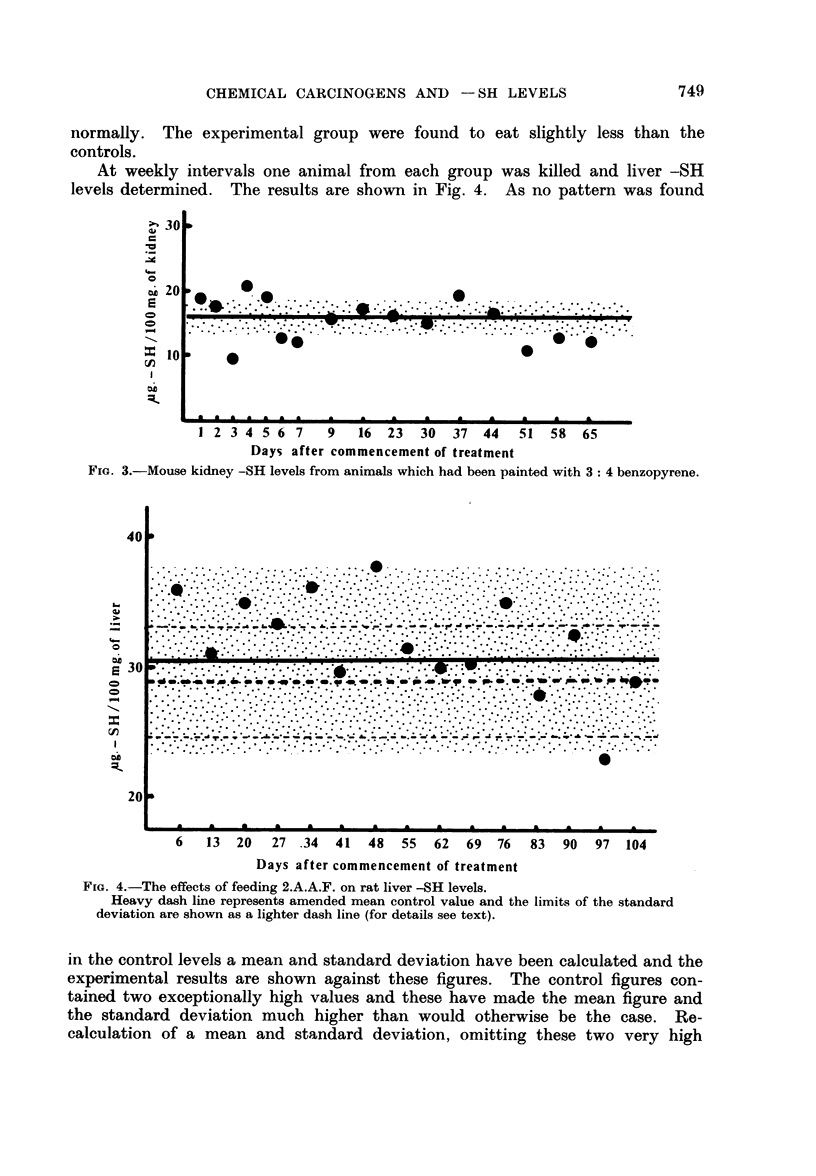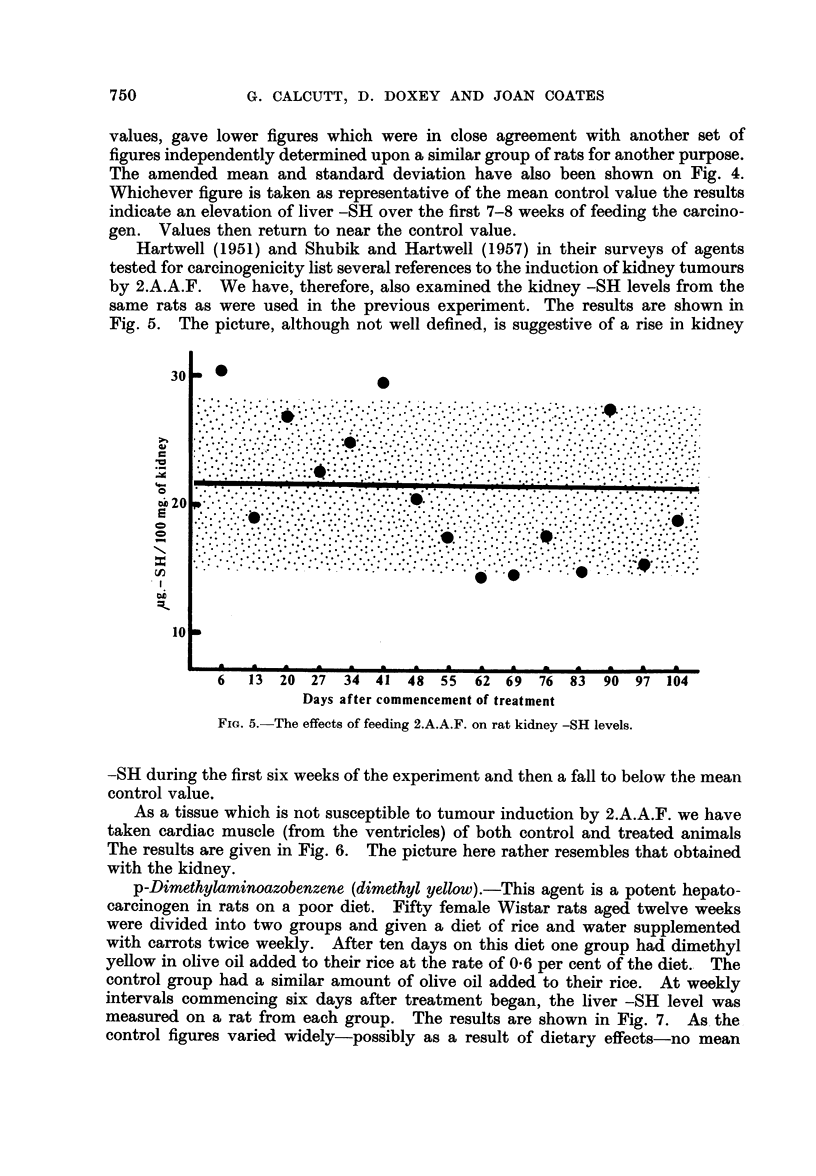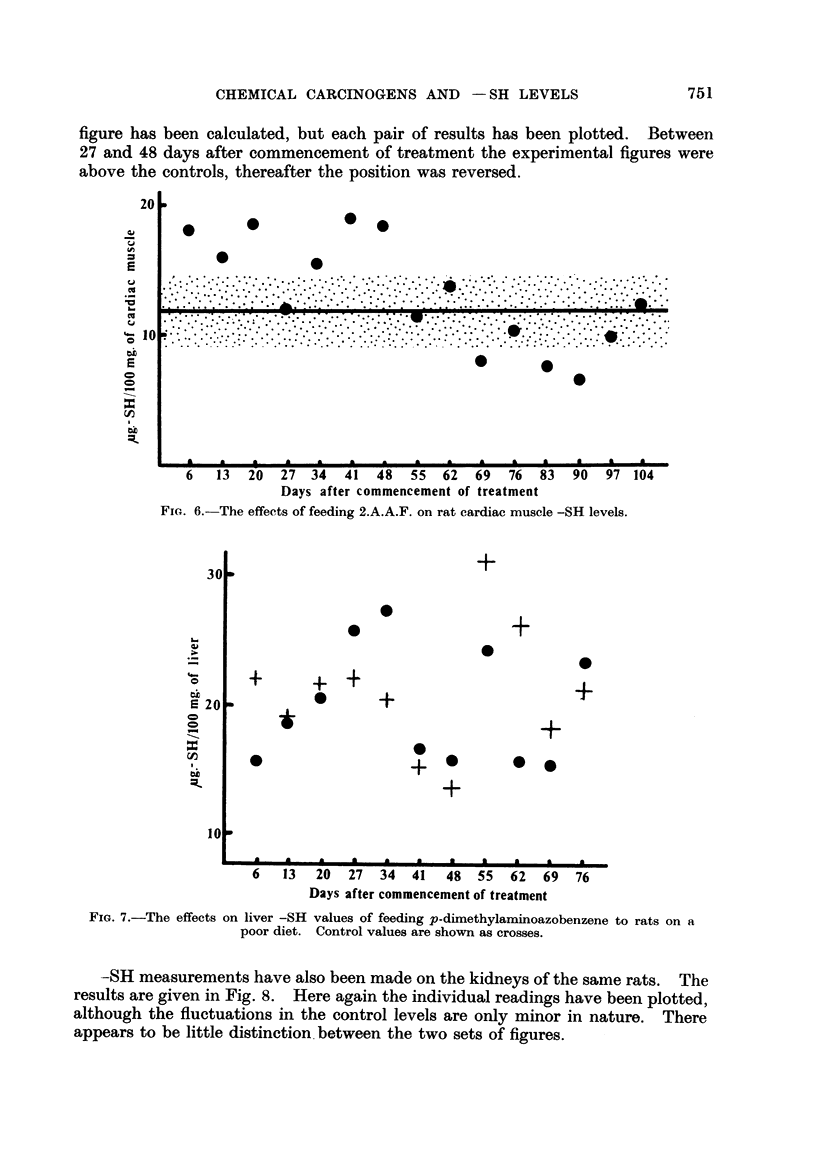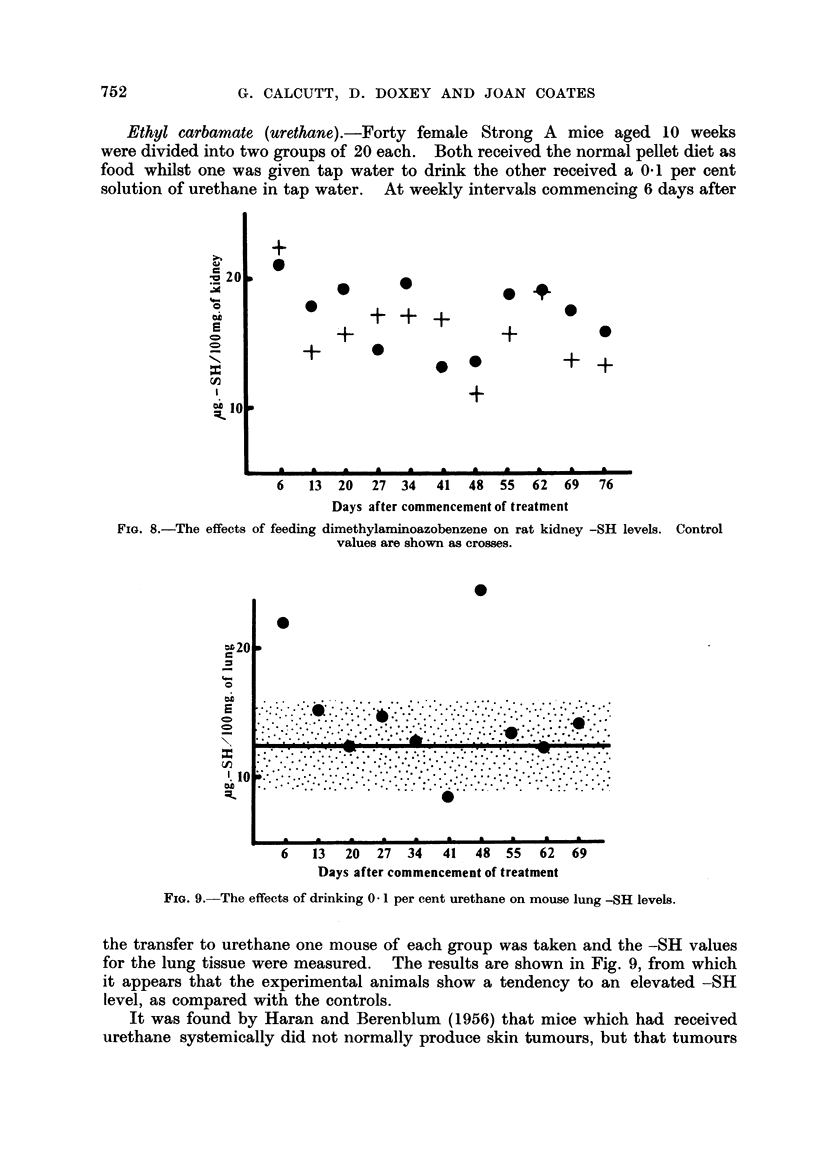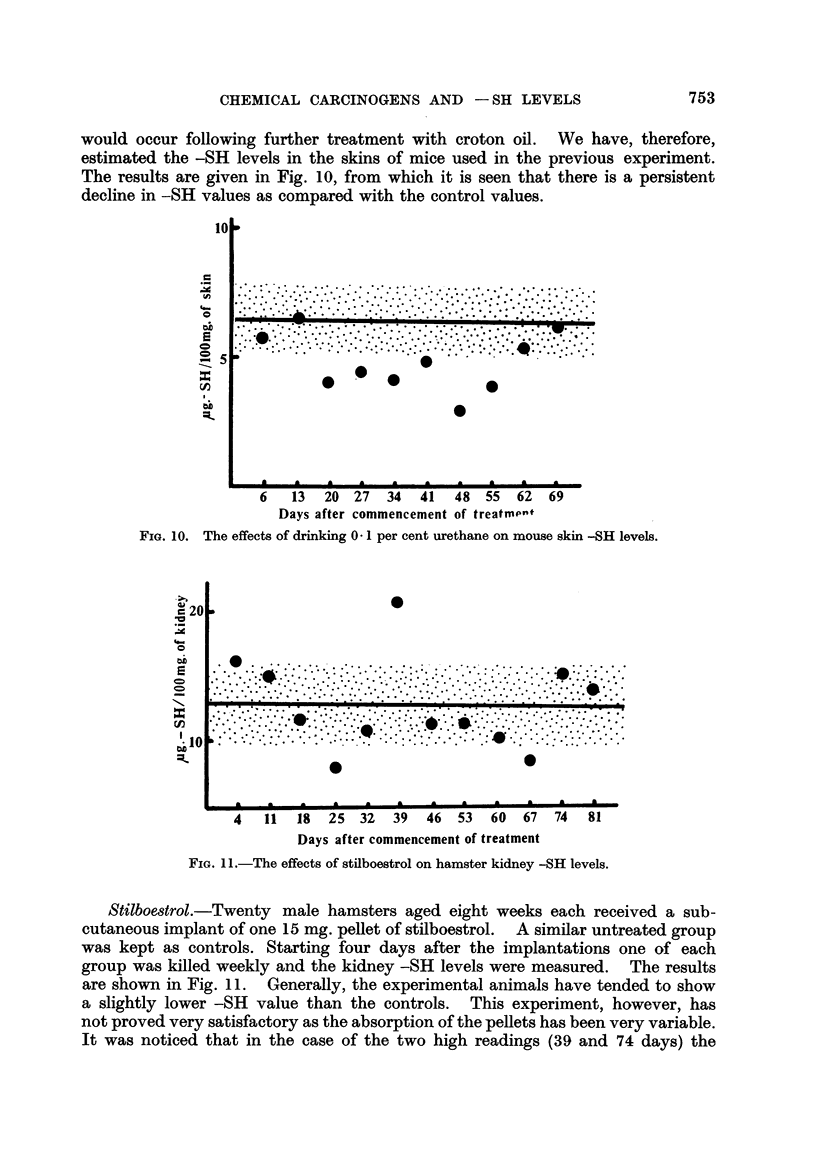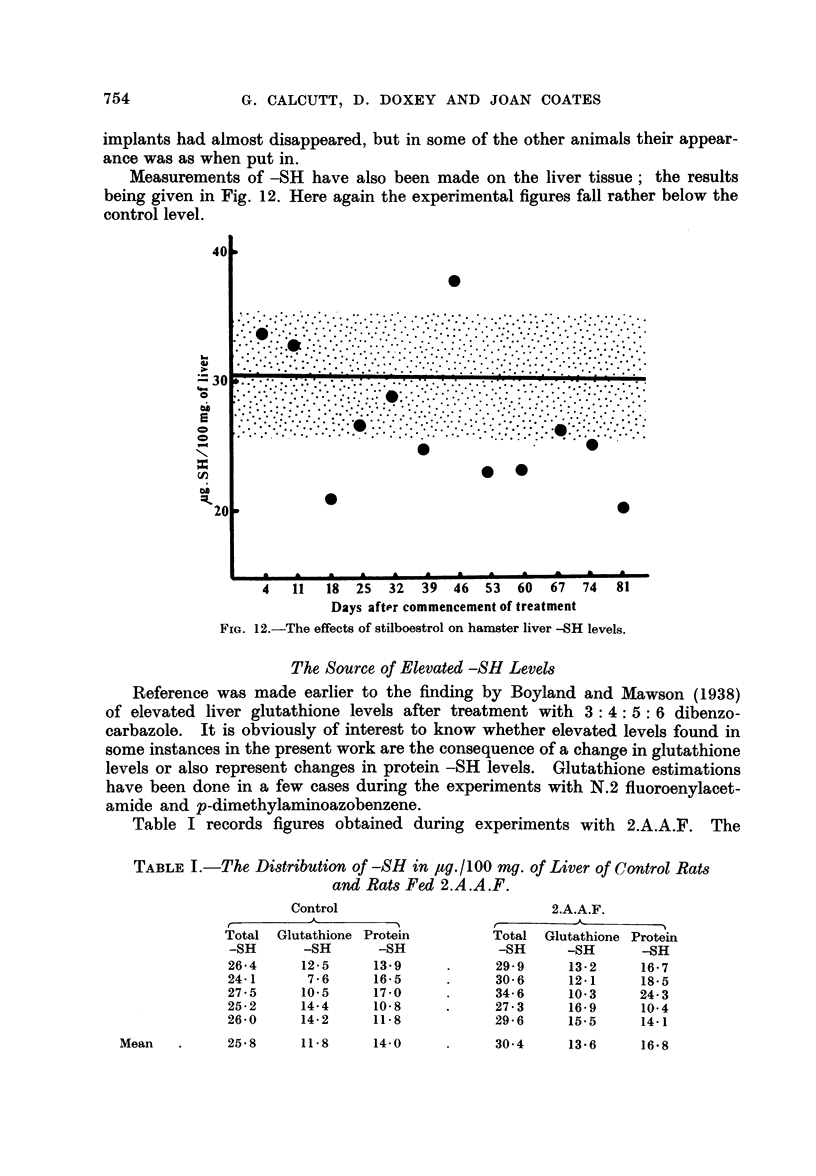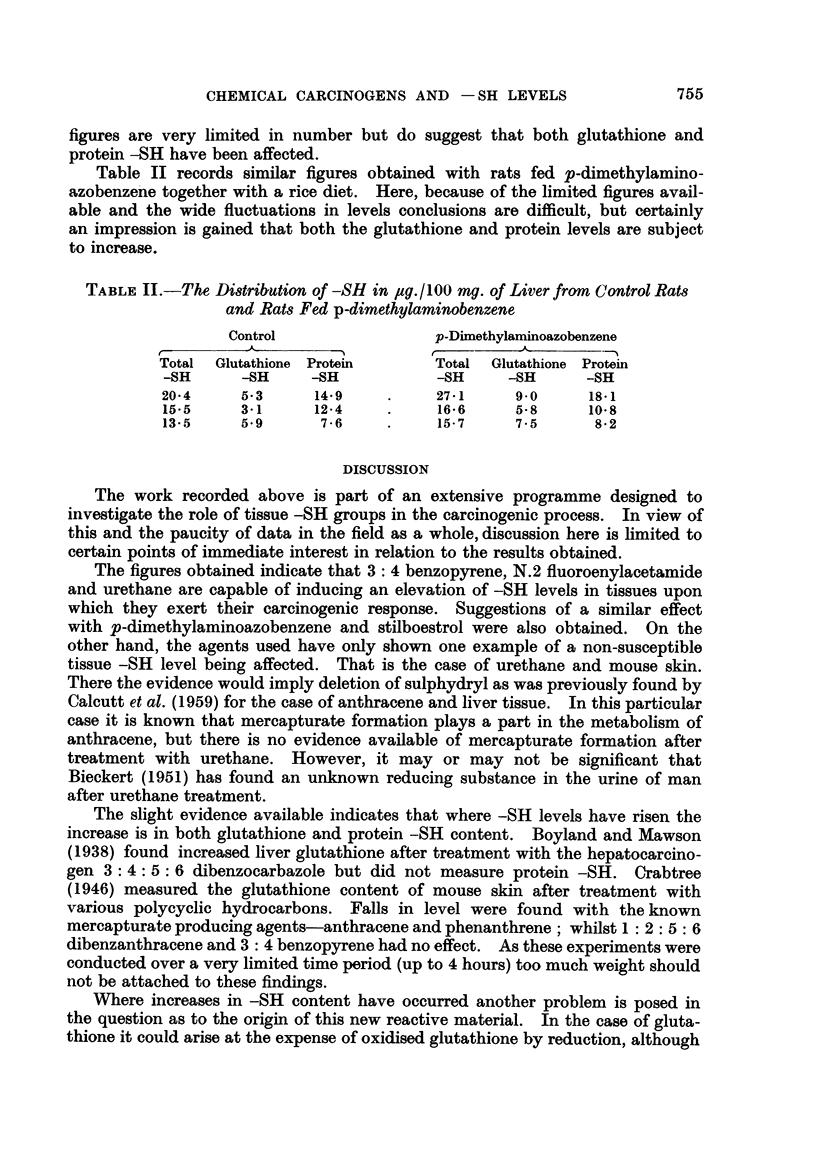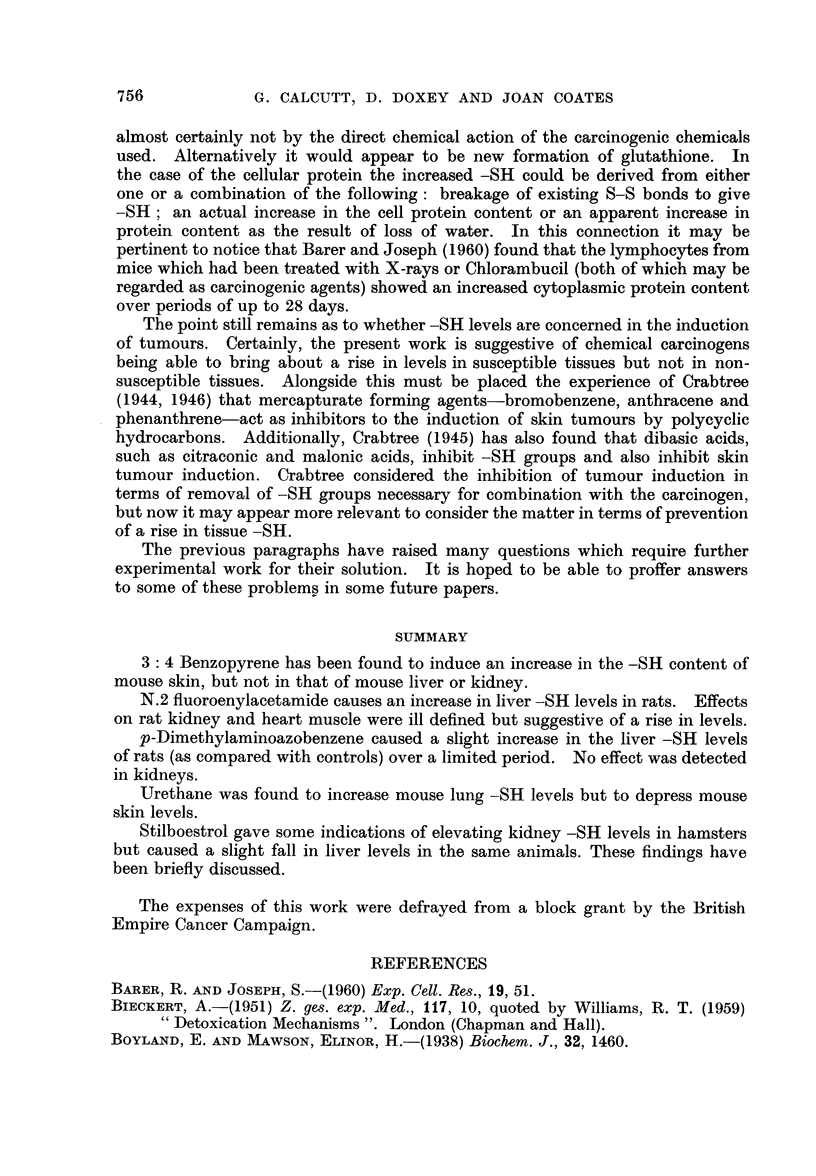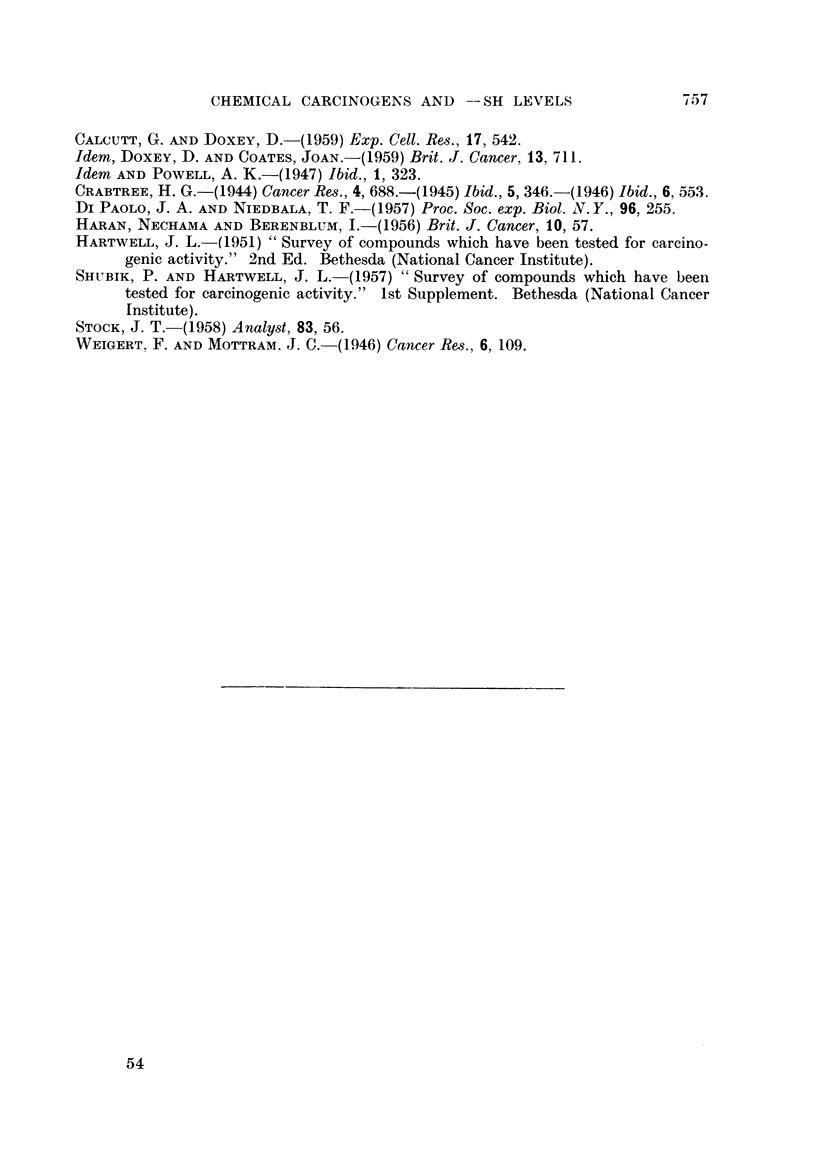# The Effects of Some Chemical Carcinogens upon the -SH Levels of Target and Non-Target Tissues

**DOI:** 10.1038/bjc.1960.85

**Published:** 1960-12

**Authors:** G. Calcutt, D. Doxey, Joan Coates


### THE EFFECTS OF SOME CHEMICAL CARCINOGENS UPON THE –SH LEVELS OF TARGET AND NON-TARGET TISSUES

G. CALCUTT, D. DOXEY AND JOAN COATES

*From the Department of Cancer Research, Mount Vernon Hospital, and the Radium Institute, Northwood, Middlesex*

Received for publication October 29, 1960

THE role of sulphydryl (–SH) groups in both tumour growth and the induction of tumours has been a subject of interest for many years. Actual experimental work in this field has been limited and often only via indirect approaches. Methods of measuring the –SH within tissues have been unsatisfactory and in many cases involved tedious procedures with specialised equipment. Recently, Calcutt and Doxey (1959) described a simple technique for tissue –SH measurements using small weights of tissue. This technique was then used to determine the effects of various carcinogenic and non-carcinogenic hydrocarbons on the liver –SH values of mice previously treated with one of these hydrocarbons (Calcutt, Doxey and Coates, 1959). This work dealt with the relationships of –SH levels to the metabolism of the agents in question, since all are known to be metabolised in the liver but none can be regarded as a true hepato-carcinogen.

The present paper records a study of the effects of known chemical carcinogens on the –SH levels of susceptible and non-susceptible tissues. We have been influenced in this choice of subject by knowledge of two previous papers which bear upon this field. Boyland and Mawson (1938) found that intraperitoneal injection of 3 : 4 : 5 : 6 dibenzocarbazole (a known hepato-carcinogen) causes a considerable rise in liver glutathione which is persistent over several months. The livers of mice injected with methylcholanthrene or 1 : 2 : 5 : 6 dibenzanthracene (neither being a liver carcinogen) did not show a similar increase in glutathione. Di Paolo and Niedbala (1957) found that after a single painting with either 1 : 2 : 5 : 6 dibenzanthracene or 9 : 10 dimethylbenzanthracene the –SH content of mouse skin was raised above the normal value and this increase was persistent for at least five days. The chemically related, but non-carcinogenic compound, anthracene, was found to have no effect on skin –SH levels.

### *The Measurement of Tissue –SH*

As the technique used has been described in detail elsewhere, only an outline will be given here. Known weights of slices of the tissue under examination are immersed in a known volume of a standardised $5 \times 10^{-5}$ M solution of *p*-chloromercuribenzoic acid (C.M.B.). After a time interval (approximately 30 minutes) for reaction with the –SH groups to occur, the unchanged C.M.B. is titrated potentiometrically with $2 \times 10^{-4}$ M cysteine hydrochloride. The millivoltmeters originally used in developing this technique have now been replaced by transistorised titrimeters based on the design of Stock (1958). These have been found superior to millivoltmeters in use as settling times are negligible and end point deflections are large and easily read. During the present work every titration has been run in duplicate, the two runs being done at the same time on separate sets of instruments. End points on the two sets of equipment have been either completely in agreement or within 0·1 ml. of one another. The mean of the two results has been used for calculation purposes. The technique has been adapted for glutathione estimations. Weighed amounts of tissue have been ground in a few ml. of 10 per cent metaphosphoric acid and centrifuged. The supernatant together with a further washing of the tissue has then been neutralised to pH 7·0 with 5 per cent caustic soda solution using Bromo-cresol purple as an indicator. This solution has been treated for –SH measurement as in the case of tissues. The neutralisation of the acid solution of glutathione is essential as C.M.B. is insoluble in acids.

The weights of tissue used for estimations have normally been in the range 80–200 mg.

*Experimental*

3 : 4 *Benzopyrene.*—One hundred and seventy strong A male mice were divided into two groups. The animals of one group each received a once weekly application of 0·2 ml. of a 0·5 per cent acetone solution of 3 : 4 benzopyrene. The other group were used as controls. Daily for the first week and then at intervals of seven days five animals of each group were killed. Small pieces of skin (after the fur had been clipped) were removed from the painted areas or from the corresponding areas of the untreated mice. Estimations of –SH were then made on the pooled samples of skin. The results over a period of 65 days are shown in Fig. 1. Since, over this period, no pattern has been detectable in the levels for the control skins these figures have been shown as a mean figure and a standard deviation. All measurements are expressed in terms of $\mu$g. of –SH per 100 mg. of wet weight of skin.

After the first treatment with benzopyrene the –SH level of the skin rose and then declined over the first week to a normal level. Further treatment gave enhanced levels for about 6–7 weeks when a return to the normal level occurred.

It has been shown by Calcutt and Powell (1947) that after skin painting of polycyclic hydrocarbons a large amount of the applied reagent is removed by the animals licking themselves. The agent removed in this fashion is carried down the gut and is transferred to the internal organs of the body. We have, therefore, done –SH measurements on both the liver and the kidneys of both control and experimental animals. The choice of these two tissues is based on the evidence of Weigert and Mottram (1946) that benzopyrene is metabolised in both, but that there is no good evidence for either being susceptible to the carcinogenic activity of 3 : 4 benzopyrene.

In Fig. 2 the results obtained with liver are shown. No definite indications of any well defined changes were obtained. The kidney results are shown in Fig. 3. Here again there was no evidence of specific changes in –SH levels.

*N*–2 *Fluoroenylacetamide* (2 *acetylaminofluorene* ; 2.*A.A.F.*).—Fifty female Wistar rats aged twelve weeks were divided into two equal groups. Both groups were fed a diet prepared by grinding up a normal pelletted food and making this up with water to give a firm mash. The carcinogen, 2.A.A.F., was added at the rate of 0·4 per cent to the diet of one group. Food prepared in this fashion was found to be acceptable to the animals and the control group ate and grew as

FIG. 1.—The effects of treatment with 3 : 4 benzopyrene on mouse skin -SH levels.
In this and all further figures, the mean control value is shown as a heavy line and the standard deviation by the dotted area. Experimental figures are shown as filled circles.

FIG. 2.—Mouse liver -SH values from animals which had been painted with 3 : 4 benzopyrene.

normally. The experimental group were found to eat slightly less than the controls.

At weekly intervals one animal from each group was killed and liver –SH levels determined. The results are shown in Fig. 4. As no pattern was found

FIG. 3.—Mouse kidney –SH levels from animals which had been painted with 3 : 4 benzopyrene.

FIG. 4.—The effects of feeding 2.A.A.F. on rat liver –SH levels.
Heavy dash line represents amended mean control value and the limits of the standard deviation are shown as a lighter dash line (for details see text).

in the control levels a mean and standard deviation have been calculated and the experimental results are shown against these figures. The control figures contained two exceptionally high values and these have made the mean figure and the standard deviation much higher than would otherwise be the case. Recalculation of a mean and standard deviation, omitting these two very high values, gave lower figures which were in close agreement with another set of figures independently determined upon a similar group of rats for another purpose. The amended mean and standard deviation have also been shown on Fig. 4. Whichever figure is taken as representative of the mean control value the results indicate an elevation of liver –SH over the first 7–8 weeks of feeding the carcinogen. Values then return to near the control value.

Hartwell (1951) and Shubik and Hartwell (1957) in their surveys of agents tested for carcinogenicity list several references to the induction of kidney tumours by 2.A.A.F. We have, therefore, also examined the kidney –SH levels from the same rats as were used in the previous experiment. The results are shown in Fig. 5. The picture, although not well defined, is suggestive of a rise in kidney

FIG. 5.—The effects of feeding 2.A.A.F. on rat kidney –SH levels.

–SH during the first six weeks of the experiment and then a fall to below the mean control value.

As a tissue which is not susceptible to tumour induction by 2.A.A.F. we have taken cardiac muscle (from the ventricles) of both control and treated animals The results are given in Fig. 6. The picture here rather resembles that obtained with the kidney.

p-*Dimethylaminoazobenzene* (*dimethyl yellow*).—This agent is a potent hepatocarcinogen in rats on a poor diet. Fifty female Wistar rats aged twelve weeks were divided into two groups and given a diet of rice and water supplemented with carrots twice weekly. After ten days on this diet one group had dimethyl yellow in olive oil added to their rice at the rate of 0·6 per cent of the diet. The control group had a similar amount of olive oil added to their rice. At weekly intervals commencing six days after treatment began, the liver –SH level was measured on a rat from each group. The results are shown in Fig. 7. As the control figures varied widely—possibly as a result of dietary effects—no mean figure has been calculated, but each pair of results has been plotted. Between 27 and 48 days after commencement of treatment the experimental figures were above the controls, thereafter the position was reversed.

FIG. 6.—The effects of feeding 2.A.A.F. on rat cardiac muscle –SH levels.

FIG. 7.—The effects on liver –SH values of feeding *p*-dimethylaminoazobenzene to rats on a poor diet. Control values are shown as crosses.

–SH measurements have also been made on the kidneys of the same rats. The results are given in Fig. 8. Here again the individual readings have been plotted, although the fluctuations in the control levels are only minor in nature. There appears to be little distinction between the two sets of figures.

*Ethyl carbamate* (*urethane*).—Forty female Strong A mice aged 10 weeks were divided into two groups of 20 each. Both received the normal pellet diet as food whilst one was given tap water to drink the other received a 0·1 per cent solution of urethane in tap water. At weekly intervals commencing 6 days after

FIG. 8.—The effects of feeding dimethylaminoazobenzene on rat kidney –SH levels. Control values are shown as crosses.

FIG. 9.—The effects of drinking 0·1 per cent urethane on mouse lung –SH levels.

the transfer to urethane one mouse of each group was taken and the –SH values for the lung tissue were measured. The results are shown in Fig. 9, from which it appears that the experimental animals show a tendency to an elevated –SH level, as compared with the controls.

It was found by Haran and Berenblum (1956) that mice which had received urethane systemically did not normally produce skin tumours, but that tumours would occur following further treatment with croton oil. We have, therefore, estimated the –SH levels in the skins of mice used in the previous experiment. The results are given in Fig. 10, from which it is seen that there is a persistent decline in –SH values as compared with the control values.

FIG. 10. The effects of drinking 0·1 per cent urethane on mouse skin –SH levels.

FIG. 11.—The effects of stilboestrol on hamster kidney –SH levels.

*Stilboestrol.*—Twenty male hamsters aged eight weeks each received a subcutaneous implant of one 15 mg. pellet of stilboestrol. A similar untreated group was kept as controls. Starting four days after the implantations one of each group was killed weekly and the kidney –SH levels were measured. The results are shown in Fig. 11. Generally, the experimental animals have tended to show a slightly lower –SH value than the controls. This experiment, however, has not proved very satisfactory as the absorption of the pellets has been very variable. It was noticed that in the case of the two high readings (39 and 74 days) the implants had almost disappeared, but in some of the other animals their appearance was as when put in.

Measurements of –SH have also been made on the liver tissue ; the results being given in Fig. 12. Here again the experimental figures fall rather below the control level.

Fig. 12.—The effects of stilboestrol on hamster liver –SH levels.

*The Source of Elevated –SH Levels*

Reference was made earlier to the finding by Boyland and Mawson (1938) of elevated liver glutathione levels after treatment with 3 : 4 : 5 : 6 dibenzocarbazole. It is obviously of interest to know whether elevated levels found in some instances in the present work are the consequence of a change in glutathione levels or also represent changes in protein –SH levels. Glutathione estimations have been done in a few cases during the experiments with N.2 fluoroenylacetamide and *p*-dimethylaminoazobenzene.

Table I records figures obtained during experiments with 2.A.A.F. The

Table I.—*The Distribution of –SH in μg./100 mg. of Liver of Control Rats and Rats Fed 2.A.A.F.*

| | Control | | | 2.A.A.F. | | |
|---|---|---|---|---|---|---|
| | Total –SH | Glutathione –SH | Protein –SH | Total –SH | Glutathione –SH | Protein –SH |
| | 26·4 | 12·5 | 13·9 | 29·9 | 13·2 | 16·7 |
| | 24·1 | 7·6 | 16·5 | 30·6 | 12·1 | 18·5 |
| | 27·5 | 10·5 | 17·0 | 34·6 | 10·3 | 24·3 |
| | 25·2 | 14·4 | 10·8 | 27·3 | 16·9 | 10·4 |
| | 26·0 | 14·2 | 11·8 | 29·6 | 15·5 | 14·1 |
| Mean | 25·8 | 11·8 | 14·0 | 30·4 | 13·6 | 16·8 |

figures are very limited in number but do suggest that both glutathione and protein –SH have been affected.

Table II records similar figures obtained with rats fed *p*-dimethylamino-azobenzene together with a rice diet. Here, because of the limited figures available and the wide fluctuations in levels conclusions are difficult, but certainly an impression is gained that both the glutathione and protein levels are subject to increase.

TABLE II.—*The Distribution of –SH in μg./100 mg. of Liver from Control Rats and Rats Fed* p-*dimethylaminobenzene*

| Control | | | | *p*-Dimethylaminoazobenzene | | |
|---|---|---|---|---|---|---|
| Total –SH | Glutathione –SH | Protein –SH | | Total –SH | Glutathione –SH | Protein –SH |
| 20·4 | 5·3 | 14·9 | . | 27·1 | 9·0 | 18·1 |
| 15·5 | 3·1 | 12·4 | . | 16·6 | 5·8 | 10·8 |
| 13·5 | 5·9 | 7·6 | . | 15·7 | 7·5 | 8·2 |

### DISCUSSION

The work recorded above is part of an extensive programme designed to investigate the role of tissue –SH groups in the carcinogenic process. In view of this and the paucity of data in the field as a whole, discussion here is limited to certain points of immediate interest in relation to the results obtained.

The figures obtained indicate that 3 : 4 benzopyrene, N.2 fluoroenylacetamide and urethane are capable of inducing an elevation of –SH levels in tissues upon which they exert their carcinogenic response. Suggestions of a similar effect with *p*-dimethylaminoazobenzene and stilboestrol were also obtained. On the other hand, the agents used have only shown one example of a non-susceptible tissue –SH level being affected. That is the case of urethane and mouse skin. There the evidence would imply deletion of sulphydryl as was previously found by Calcutt *et al.* (1959) for the case of anthracene and liver tissue. In this particular case it is known that mercapturate formation plays a part in the metabolism of anthracene, but there is no evidence available of mercapturate formation after treatment with urethane. However, it may or may not be significant that Bieckert (1951) has found an unknown reducing substance in the urine of man after urethane treatment.

The slight evidence available indicates that where –SH levels have risen the increase is in both glutathione and protein –SH content. Boyland and Mawson (1938) found increased liver glutathione after treatment with the hepatocarcinogen 3 : 4 : 5 : 6 dibenzocarbazole but did not measure protein –SH. Crabtree (1946) measured the glutathione content of mouse skin after treatment with various polycyclic hydrocarbons. Falls in level were found with the known mercapturate producing agents—anthracene and phenanthrene ; whilst 1 : 2 : 5 : 6 dibenzanthracene and 3 : 4 benzopyrene had no effect. As these experiments were conducted over a very limited time period (up to 4 hours) too much weight should not be attached to these findings.

Where increases in –SH content have occurred another problem is posed in the question as to the origin of this new reactive material. In the case of glutathione it could arise at the expense of oxidised glutathione by reduction, although almost certainly not by the direct chemical action of the carcinogenic chemicals used. Alternatively it would appear to be new formation of glutathione. In the case of the cellular protein the increased –SH could be derived from either one or a combination of the following : breakage of existing S–S bonds to give –SH ; an actual increase in the cell protein content or an apparent increase in protein content as the result of loss of water. In this connection it may be pertinent to notice that Barer and Joseph (1960) found that the lymphocytes from mice which had been treated with X-rays or Chlorambucil (both of which may be regarded as carcinogenic agents) showed an increased cytoplasmic protein content over periods of up to 28 days.

The point still remains as to whether –SH levels are concerned in the induction of tumours. Certainly, the present work is suggestive of chemical carcinogens being able to bring about a rise in levels in susceptible tissues but not in non-susceptible tissues. Alongside this must be placed the experience of Crabtree (1944, 1946) that mercapturate forming agents—bromobenzene, anthracene and phenanthrene—act as inhibitors to the induction of skin tumours by polycyclic hydrocarbons. Additionally, Crabtree (1945) has also found that dibasic acids, such as citraconic and malonic acids, inhibit –SH groups and also inhibit skin tumour induction. Crabtree considered the inhibition of tumour induction in terms of removal of –SH groups necessary for combination with the carcinogen, but now it may appear more relevant to consider the matter in terms of prevention of a rise in tissue –SH.

The previous paragraphs have raised many questions which require further experimental work for their solution. It is hoped to be able to proffer answers to some of these problems in some future papers.

SUMMARY

3 : 4 Benzopyrene has been found to induce an increase in the –SH content of mouse skin, but not in that of mouse liver or kidney.

N.2 fluoroenylacetamide causes an increase in liver –SH levels in rats. Effects on rat kidney and heart muscle were ill defined but suggestive of a rise in levels.

*p*-Dimethylaminoazobenzene caused a slight increase in the liver –SH levels of rats (as compared with controls) over a limited period. No effect was detected in kidneys.

Urethane was found to increase mouse lung –SH levels but to depress mouse skin levels.

Stilboestrol gave some indications of elevating kidney –SH levels in hamsters but caused a slight fall in liver levels in the same animals. These findings have been briefly discussed.

The expenses of this work were defrayed from a block grant by the British Empire Cancer Campaign.

REFERENCES

BARER, R. AND JOSEPH, S.—(1960) *Exp. Cell. Res.*, **19**, 51.
BIECKERT, A.—(1951) *Z. ges. exp. Med.*, **117**, 10, quoted by Williams, R. T. (1959) " Detoxication Mechanisms ". London (Chapman and Hall).
BOYLAND, E. AND MAWSON, ELINOR, H.—(1938) *Biochem. J.*, **32**, 1460.

CALCUTT, G. AND DOXEY, D.—(1959) *Exp. Cell. Res.*, **17**, 542.
*Idem*, DOXEY, D. AND COATES, JOAN.—(1959) *Brit. J. Cancer*, **13**, 711.
*Idem* AND POWELL, A. K.—(1947) *Ibid.*, **1**, 323.
CRABTREE, H. G.—(1944) *Cancer Res.*, **4**, 688.—(1945) *Ibid.*, **5**, 346.—(1946) *Ibid.*, **6**, 553.
DI PAOLO, J. A. AND NIEDBALA, T. F.—(1957) *Proc. Soc. exp. Biol. N.Y.*, **96**, 255.
HARAN, NECHAMA AND BERENBLUM, I.—(1956) *Brit. J. Cancer*, **10**, 57.
HARTWELL, J. L.—(1951) "Survey of compounds which have been tested for carcinogenic activity." 2nd Ed. Bethesda (National Cancer Institute).
SHUBIK, P. AND HARTWELL, J. L.—(1957) "Survey of compounds which have been tested for carcinogenic activity." 1st Supplement. Bethesda (National Cancer Institute).
STOCK, J. T.—(1958) *Analyst*, **83**, 56.
WEIGERT, F. AND MOTTRAM, J. C.—(1946) *Cancer Res.*, **6**, 109.